# Potential of *Aedes aegypti* populations in Madeira Island to transmit dengue and chikungunya viruses

**DOI:** 10.1186/s13071-018-3081-4

**Published:** 2018-09-12

**Authors:** Gonçalo Seixas, Henri Jupille, Pei-Shi Yen, Bela Viveiros, Anna-Bella Failloux, Carla Alexandra Sousa

**Affiliations:** 1 Global Health and Tropical Medicine, Instituto de Higiene e Medicina Tropical, Universidade Nova de Lisboa, Lisboa, Portugal; 2 Arboviruses and Insect Vectors, Department of Virology, Institut Pasteur, Paris, France; 3 Departamento de Planeamento, Saúde e Administração Geral do Instituto de Administração da Saúde e Assuntos Sociais, IP-RAM, Funchal, Madeira Portugal

**Keywords:** Arbovirus, Europe, Vector competence, *Aedes aegypti*

## Abstract

**Background:**

*Aedes* spp. mosquitoes mainly transmit the arboviruses dengue virus (DENV) and chikungunya virus (CHIKV) in urban areas, causing a severe public health problem. In 2012–2013, a major dengue outbreak occurred on Madeira Island where the mosquito *Aedes aegypti* was the only vector. Up to now, the competence of *Ae. aegypti* populations from Madeira to transmit DENV or CHIKV remains unknown. This study aimed to assess experimentally the ability of *Ae. aegypti* populations from Madeira to transmit these viruses.

**Results:**

By orally exposing mosquitoes to CHIKV (NC/2011-568) and DENV-2 (Bangkok), the vector competence of two field-collected *Ae. aegypti* populations, i.e. Funchal and Paúl do Mar, was evaluated. We found that both populations were similarly infected and ensured the dissemination and transmission of CHIKV at the same rates. With DENV-2, viral dissemination was significantly higher in the Funchal population compared to Paúl do Mar. We found no significant differences in transmission rates between populations.

**Conclusions:**

To our knowledge, this study has demonstrated for the first time the ability of temperate European *Ae. aegypti* populations from Madeira to transmit DENV and CHIKV. As our results suggest, there is a potential risk for the local transmission of DENV and CHIKV if introduced to Madeira or continental Europe where *Aedes albopictus* is present. Our results highlight the need for continuing vector surveillance and control on Madeira Island to future-proof the Island against mosquito-borne epidemics.

## Background

*Aedes aegypti* (Linnaeus, 1762) is known to be the vector of several arboviruses [1]. While originally native to Africa, this species has continuously expanded its range during the last centuries [2], including to the European territories such as Madeira Island (Portugal), Georgia and occasionally in the Netherlands [3, 4]. First detected in 2005 in the city of Funchal on Madeira, this mosquito is now widely distributed throughout the southern coast of the island [5] and was responsible for a major dengue outbreak in October 2012 with thousands of dengue cases [6], representing the first autochthonous cases in a Portuguese territory.

Beside the dengue virus (DENV; genus *Flavivirus*, family *Flaviviridae*), *Ae. aegypti* is also experimentally competent for chikungunya virus (CHIKV; genus *Alphavirus*, family *Togaviridae*) [7, 8]. Dengue and chikungunya are serious public health issues in tropical regions, and each virus family has different serotypes, lineages and genotypes [9, 10]. Dengue is caused by four genetically distinct DENV serotypes (1, 2, 3 and 4) and generally lead to a self-limited febrile illness characterised by a headache, fever and rash. CHIKV causes an acute febrile illness characterised by severe arthralgia [11]. Phylogenetic analysis suggests that CHIKV lineages can be classified into three distinct genotypes: Asian, West African and Eastern/Central/Southern African (ECSA). Both DENV and CHIKV infections have a large proportion of asymptomatic cases contributing actively to virus dissemination and transmission [12].

The recent emergence of dengue and chikungunya in Europe, such as the 2012 outbreaks of dengue in Madeira and chikungunya in France [13, 14] and Italy [15], have raised concerns of arbovirus transmission in countries infested by mosquito species that could sustain epidemics, especially *Ae. aegypti* and/or *Aedes albopictus* (Skuse, 1894) [16]. Due to the intense social and commercial relations with Brazil and Venezuela, Madeira Island could serve as a source for the introduction of *Ae. aegypti* and/or arboviruses to continental Europe [17]. The risk of arboviral outbreaks in Madeira is real since imported cases of DENV and Zika virus (ZIKV) were detected in citizens returning from DENV- and ZIKV-infected countries [18] and local *Ae. aegypti* populations from Madeira were experimentally susceptible to ZIKV [19]. This study aims to assess the ability of *Ae. aegypti* populations from Madeira Island to experimentally transmit CHIKV and DENV. The results obtained will provide a solid basis for decisions regarding disease prevention and control for the Madeira Health Authorities and decision-makers in Europe.

## Methods

### Mosquitoes

Two *Ae. aegypti* populations from Madeira were used in vector competence assays: the Funchal population, collected in the major urban area and island’s capital city, and Paúl do Mar population collected in the most western point of the species distribution on the island, *c.*42 km away from Funchal, and considered a rural area. Mosquito eggs were collected in 2014 using widely distributed ovitraps [20] and hatched in insectaries. The larvae were split as 200–300 individuals per pan and fed with yeast tablets. Emerging adults were maintained in cages at 28 ± 1 °C with a 14 h light/10 h dark photocycle, 80% relative humidity, and supplied with a 10% sucrose solution *ad libitum*. The F1 generation was used for experimental infections.

### Viral strains

CHIKV (NC/2011-568) was isolated in 2011 from a patient by the Institut Pasteur of New Caledonia (kindly provided by Dr Myrielle Dupont-Rouzeyrol); this isolate belongs to the Asian genotype and possesses an alanine at base position 226 in the E1 envelope glycoprotein (GenBank: HE806461). DENV belonging to serotype 2 (DENV-2) was isolated in 1974 from a patient in Bangkok, Thailand [21]. Both viral stocks were produced following 2–3 passages on C6/36 *Ae. albopictus-*derived cells.

### Mosquito oral infections

Four batches of 60 one-week-old female adults were fed on an infectious blood-meal that consisted of 1400 μl of washed rabbit erythrocytes, 700 μl of viral suspension, supplemented with 5 mM adenosine triphosphate (ATP), a phagostimulant. Two feeders were prepared per virus, and a feeder was available to two batches of mosquito (successively) for 20 min. The viral titre of the infectious blood-meal was determined at 2 × 10^7^ focus-forming units (ffu)/ml for DENV-2 and 2 × 10^7^ ffu/ml for CHIKV. After exposure, fully engorged females were transferred to cardboard containers and maintained with 10% sucrose at 28 ± 1 °C and 80% relative humidity.

### Dissemination and transmission analysis

Twenty mosquitoes from each population were analysed at different time-points: 3, 6, 9 and 14 days post-infection (dpi) for CHIKV and 7 and 14 dpi for DENV-2. To estimate the infection and dissemination, the virus in bodies (including thorax and abdomen) and heads was analysed, respectively. Mosquito samples were grounded in 300 μl of Leibovitz L15 medium (Invitrogen, Carlsbad, USA) supplemented with 3% fetal bovine serum (FBS). Samples were then centrifuged for 5 min at 10,000× *rpm*, and the supernatant obtained was used for virus quantification. To estimate transmission, saliva was collected from each mosquito as previously described [22]. Briefly, legs and wings were removed from each mosquito, and the proboscis was inserted into a 20 μl tip containing 5 μl of FBS. After 20 min, saliva containing FBS was expelled into 45 μl of Leibovitz L15 medium for titration. Infection rate (IR) was used as a measure of susceptibility to each virus and corresponds to the number of mosquitoes with the infected body among the tested ones. The percentage of mosquitoes with infected heads among mosquitoes with an infected body is the dissemination rate (DR). The transmission rate (TR) is defined as the percentage of mosquitoes with infectious saliva among mosquitoes with positive viral dissemination. The number of viral particles per saliva and head was determined by titration using focus fluorescent assay on C6/36 cells. Briefly, 10-fold serial dilutions were performed for each sample and inoculated onto C6/36 cell culture in 96-well plates. After incubation at 28 °C during three days (CHIKV) or 5 days (DENV), plates were stained using hyper-immune ascetic fluid specific to CHIKV or DENV as the primary antibody. Alexa Fluor 488 goat anti-mouse IgG was used as the second antibody (Life Technologies, Carlsbad, USA).

### Statistical analysis

Statistical analyses were performed with GraphPad Prism v 6.03. Proportions were compared using Chi-square test and sample distributions with the Mann-Whitney test (*n* = 2) or Kruskal-Wallis test (*n* > 2). *P*-values > 0.05 were considered non-significant.

## Results

### *Aedes aegypti* from Madeira Island is highly susceptible to CHIKV infection

The susceptibility of *Ae. aegypti* from Madeira Island for CHIKV was studied using a viral strain belonging to Asian lineage, as the current circulating lineage in the Americas [10]. Our results showed that local *Ae. aegypti* can transmit CHIKV very efficiently: Funchal and Paúl do Mar populations were both highly susceptible to CHIKV infection, with similar infection rates [Chi-square test: *P* > 0.05; 3 dpi (*χ*^2^ = 1.02, *df* = 1, *P* = 0.31); 6 dpi (*χ*^2^ = 1.02, *df* = 1, *P* = 0.31)] ranging from 95 to 100% after 3 dpi (Table 1).

**Table 1:** Infection, dissemination and transmission rates (in %) estimated at different days after exposure of *Ae. aegypti* from Madeira to CHIKV NC/2011-568 strain

Days post-infection	Funchal			Paúl do Mar		
	IR *n* (%)	DR *n* (%)	TR *n* (%)	IR *n* (%)	DR *n* (%)	TR *n* (%)
3	95 (20)	84.2 (19)	25 (16)	100 (20)	90 (20)	27.7 (18)
6	95 (20)	94.7 (19)	33.3 (18)	100 (20)	100 (20)	55 (20)
9	100 (20)	95 (20)	57.9 (19)	100 (20)	100 (20)	50 (20)
14	100 (20)	100 (20)	40 (20)	100 (20)	100 (20)	25 (20)

*Abbreviations*: *IR* infection rate, *DR* dissemination rate, *TR* transmission rate, *n* the number of mosquitoes analysed

**Fig. 1:**
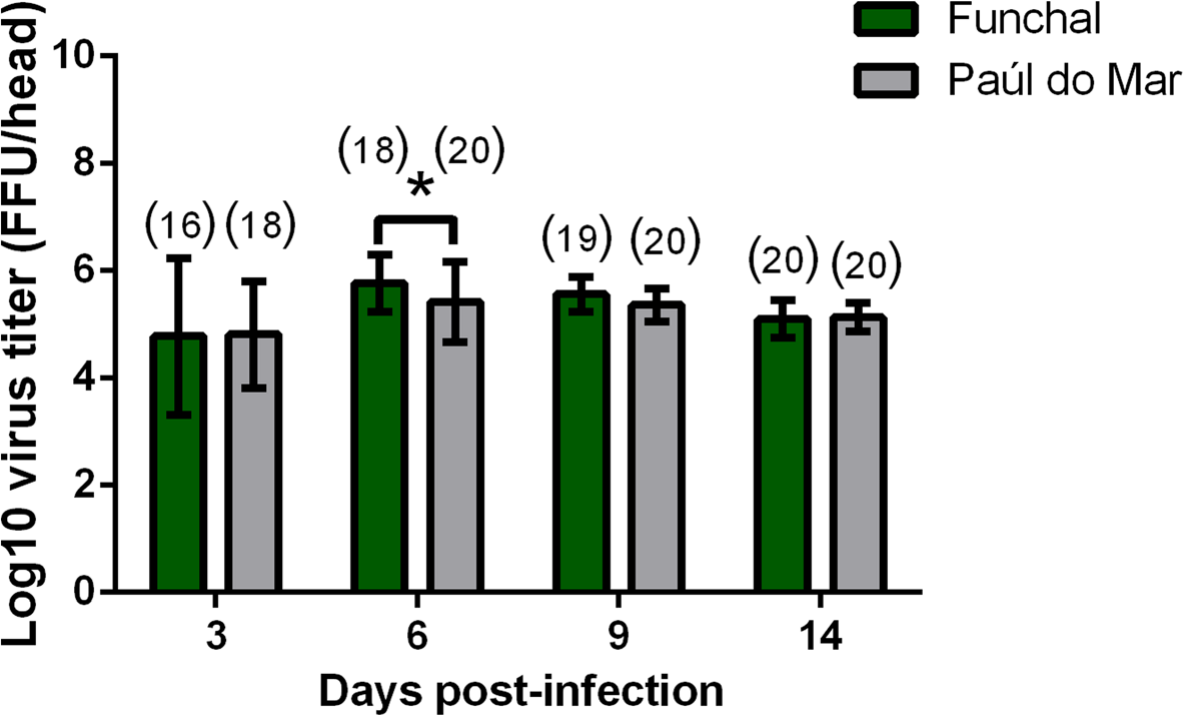
Dissemination of CHIKV in *Ae. aegypti* from Madeira Island. Mosquitoes were sacrificed, and heads were removed for viral titration at days 3, 6, 9 and 14 after infection on C6/36 cells. The numbers of analysed mosquitoes are given in parentheses. An asterisk refers to significant difference (*P*-value < 0.05). Error bars refer to the standard deviation

To measure the ability of CHIKV to cross the mosquito midgut barrier, dissemination rate (DR) was assessed at 3, 6, 9 and 14 dpi. According to the results, 100% DR was reached at 6 dpi for the Paúl do Mar population and 14 dpi for the Funchal population. No difference of DR was detected between the two populations [Chi-square test: *P* > 0.05; 3 dpi (*χ*^2^ = 0.29, *df* = 1, *P* = 0.59); 6 dpi (*χ*^2^ = 1.08, *df* = 1, *P* = 0.30); 9 dpi (*χ*^2^ = 1.02, *df* = 1, *P* = 0.31)]. The intensity of viral dissemination was evaluated by estimating the number of viral particles in head homogenates. Virus in heads was detectable from 3 dpi in both populations. The number of viral particles (Fig. 1) varied significantly throughout the time course in both populations [Kruskal-Wallis test: *P* < 0.05; Funchal (*χ*^2^ = 21.80, *df* = 3, *P* < 0.0001); Paúl do Mar (*χ*^2^ = 12.72, *df* = 3, *P* = 0.005)]. When taking into account sequential method of Bonferroni, allowing to adjust the significance level of each test to the number of tests run, both *P*-values remain significant. Significant differences were found at 6 dpi between the two populations (Mann-Whitney U-test: *Z* = 2.62, *P* = 0.009). The maximum number of CHIKV was detected at 6 dpi with 5.77 ± 0.53 log_10_ ffu/ml for the Funchal strain and 5.42 ± 0.75 log_10_ ffu/ml for the Paúl do Mar strain.

**Fig. 2:**
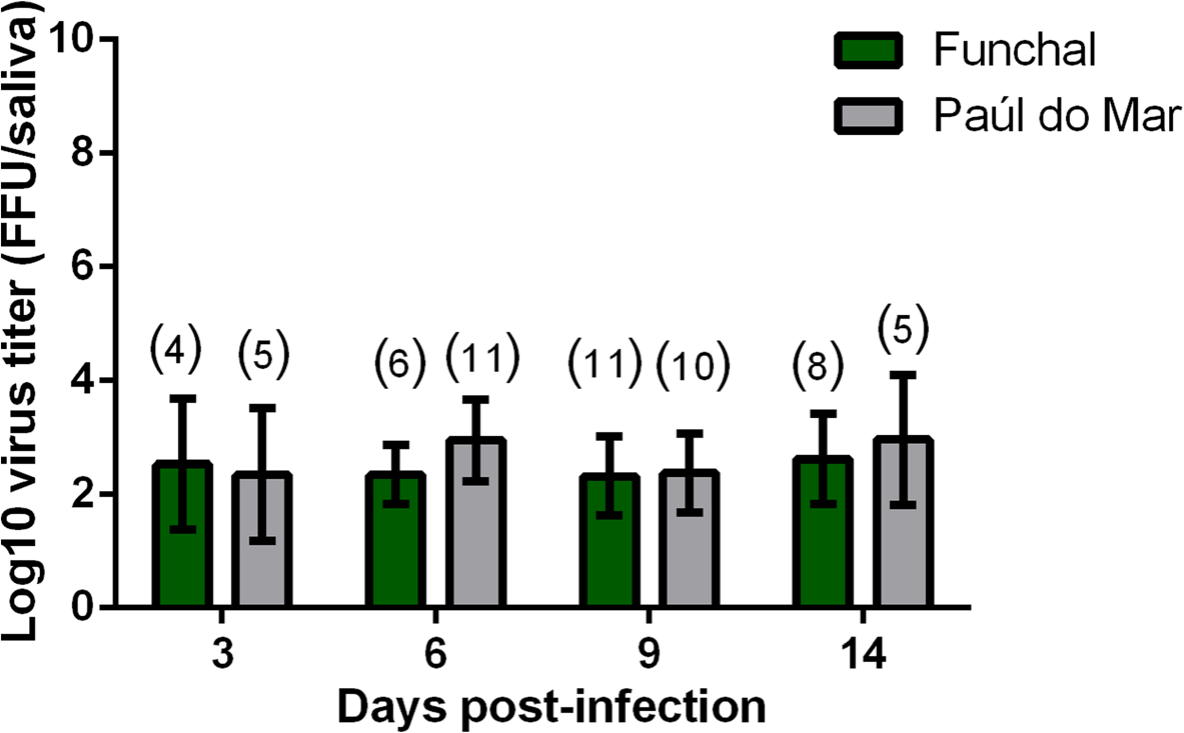
Transmission of CHIKV in the saliva of *Ae. aegypti* from Madeira Island. Mosquitoes were sacrificed, and saliva was collected individually and titrated at days 3, 6, 9 and 14 after infection on C6/36 cells. The numbers of analysed mosquitoes are given in parentheses. Error bars refer to the standard deviation

To evaluate the ability of CHIKV to reach the salivary glands and be transmitted through the mosquito bite, transmission rate (TR) was assessed at 3, 6, 9 and 14 dpi. Although only 20–25% of mosquitoes were able to transmit at 3 dpi, TR increased after 6 dpi for both populations. When comparing TR between the two populations at a given dpi, no significant differences were detected [Chi-square test: *P* > 0.05; 3 dpi (*χ*^2^ = 0.03, *df* = 1, *P* = 0.85); 6 dpi (*χ*^2^ = 1.79, *df* = 1, *P* = 0.18); 9 dpi (*χ*^2^ = 0.24, *df* = 1, *P* = 0.62); 14 dpi (*χ*^2^ = 1.02, *df* = 1, *P* = 0.31)]. The intensity of viral transmission was evaluated by quantifying the viral load in mosquito saliva. CHIKV particles reached its maximum at 14 dpi for both populations, with Funchal presenting a 2.62 ± 0.79 log_10_ ffu/ml and Paúl do Mar with 2.96 ± 1.14 log_10_ ffu/ml. At a given dpi, no significant difference was detected between populations [Mann-Whitney test: *P* > 0.05; 3 dpi (*Z* = 0.0, *P* = 1.0); 6 dpi (*Z* = -1.71, *P* = 0.09); 9 dpi (*Z* = -0.32, *P* = 0.74); 14 dpi (*Z* = -0.74, *P* = 0.46)]. In addition, the number of viral particles in saliva (Fig. 2) did not vary along with the dpi for both populations [Kruskal-Wallis test: *P* > 0.05; Funchal (*χ*^2^ = 0.98, *df* = 3, *P* = 0.80); Paúl do Mar (*χ*^2^ = 3.61, *df* = 3, *P* = 0.30)].

### *Aedes aegypti* from Funchal and Paúl do Mar transmit DENV-2 at different rates

**Fig. 3:**
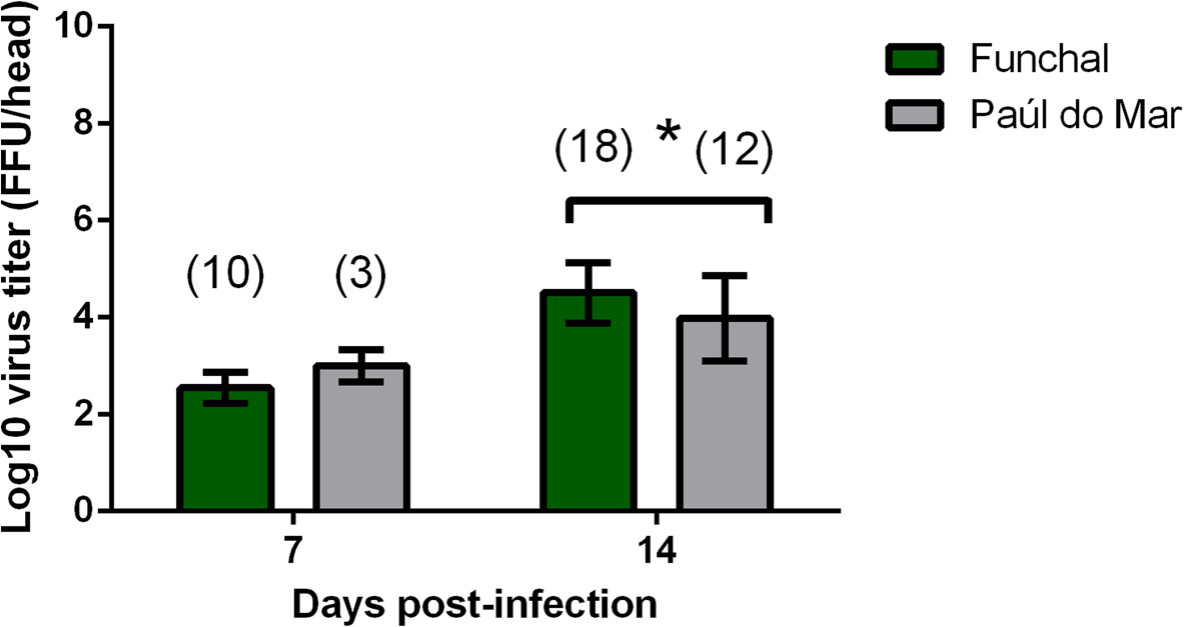
Dissemination of DENV in *Ae. aegypti* from Madeira Island. Mosquitoes were sacrificed, and heads were removed for viral titration at days 7 and 14 after infection on C6/36 cells. The numbers of analysed mosquitoes are given in parentheses. An asterisk refers to significant difference (*P*-value < 0.05). Error bars refer to the standard deviation

**Fig. 4:**
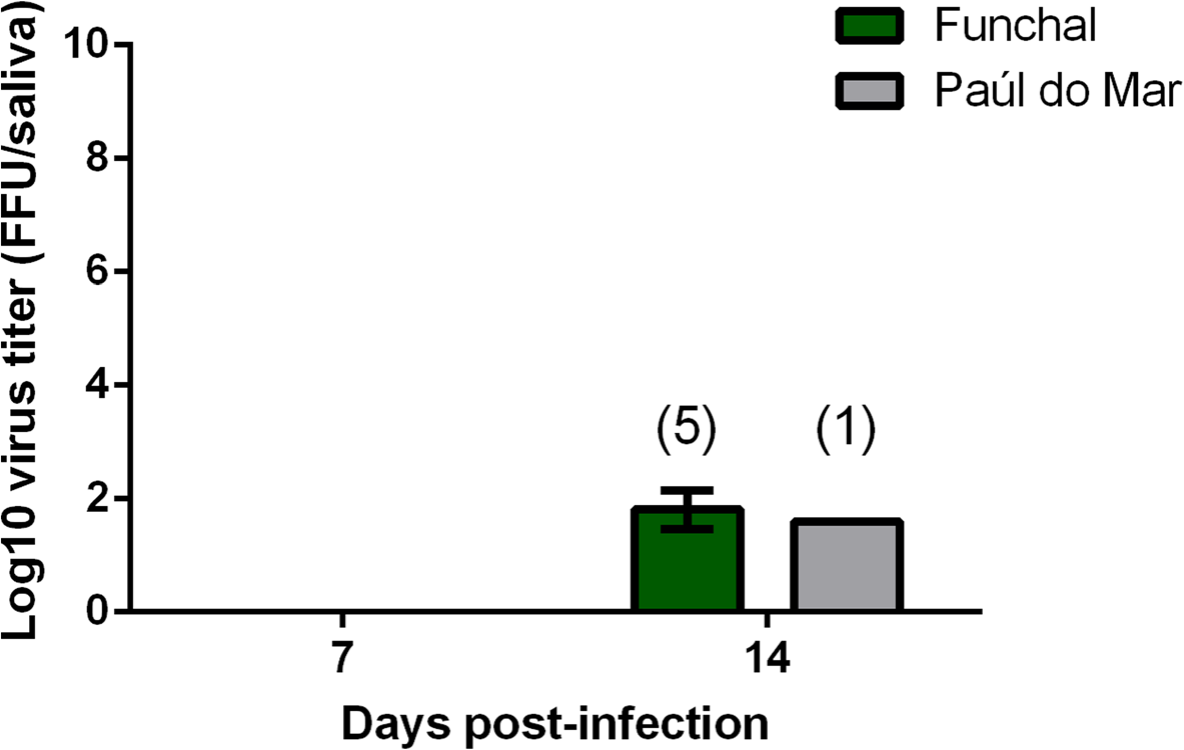
Transmission of DENV in the saliva of *Ae. aegypti* from Madeira Island. Mosquitoes were sacrificed, and saliva was collected individually and titrated at days 7 and 14 after infection on C6/36 cells. The numbers of analysed mosquitoes are given in parentheses. Error bars refer to the standard deviation

The potential of DENV-2 transmission by *Ae. aegypti* from Madeira was measured by infecting mosquitoes with a DENV-2 strain from Bangkok. IR, DR and TR were assessed at 7 and 14 dpi (Table 2). Our study indicated a different pattern of susceptibility to dengue infection compared to chikungunya infection. While both populations presented similar IR [Chi-square test *P* > 0.05; 7 dpi (*χ*^2^ = 2.05, *df* = 1, *P* = 0.15); 14 dpi (*χ*^2^ = 3.13, *df* = 1, *P* = 0.08)], the Funchal population ensured a better dissemination of DENV-2 than Paúl do Mar at 7 dpi [Chi-square test: *P* < 0.05; 7 dpi (*χ*^2^ = 4.27, *df* = 1, *P* = 0.04)] Virus titer in heads was slightly higher at 14 dpi for the Funchal strain (Mann-Whitney test: *Z* = 2.11, *P* = 0.03). The maximum number of DENV-2 particles in mosquito heads was detected at 14 dpi for both populations: 4.51 ± 0.63 log_10_ ffu/ml for Funchal population and 3.98 ± 0.88 log_10_ ffu/ml for Paúl do Mar population (Fig. 3). When examining TR, transmission with DENV-2 was lower than with CHIKV. TR reached a maximum at 14 dpi: 27.7% for the Funchal population and 8.3% for the Paúl do Mar population. No significant differences were detected between populations at each dpi (Chi-square test: *χ*^2^ = 1.70, *df* = 1, *P* = 0.19 at 14 dpi). As observed with CHIKV, the number of viral particles in saliva was lower than in heads (Figs. 3, 4). The maximum number of DENV particles in saliva was reached at 14 dpi: 1.81 ± 0.34 log_10_ ffu/ml for the Funchal population and 1.60 ffu/ml for the Paúl do Mar population (Fig. 4). Both populations presented a similar number of viral particles in saliva at 14 dpi (Mann-Whitney test: *Z* = 0.69, *P* = 0.49).

**Table 2:** Infection, dissemination and transmission rates (in %) calculated at different days after infection of *Ae. aegypti* from Madeira with DENV-2 Bangkok strain

Days post-infection	Funchal			Paúl do Mar		
	IR *n* (%)	DR *n* (%)	TR *n* (%)	IR *n* (%)	DR *n* (%)	TR *n* (%)
7	95 (20)	52.6 (19)	0 (10)	80 (20)	18.7 (16)	0 (3)
14	95 (20)	94.7 (19)	27.7 (18)	75 (20)	80 (15)	8.3 (12)

*Abbreviations*: *IR* infection rate, *DR* dissemination rate, *TR* transmission rate, *n* the number of mosquitoes analysed

## Discussion

To our knowledge, this study represents the first evaluation of the vector competence of European *Ae. aegypti* populations for the transmission of two arboviruses of medical importance, CHIKV and DENV. Since its arrival in 2005, *Ae. aegypti* has been spreading throughout Madeira Island, increasing the risk of emergence of mosquito-borne diseases. The risk became a reality in 2012 when autochthonous cases of DENV-1 were reported in Funchal [6, 23]; Venezuela in South America was the most probable origin of virus importation [24]. After this event, vector competence studies with *Ae. aegypti* from Madeira became pivotal in evaluating the risk of arboviral disease outbreak.

Our data demonstrate that the local *Ae. aegypti* populations are very susceptible to CHIKV and DENV-2 infections. Regarding CHIKV, our results showed that only three days after infection, *Ae. aegypti* from Madeira was able to transmit this virus suggesting that the extrinsic incubation period of CHIKV with this vector population was short, as expected [7]. Despite high levels of viral dissemination (84–100%), *Ae. aegypti* from both localities displayed quite similar and moderate transmission rates (25–55%), with Funchal ensuring a slightly higher virus transmission at 14 dpi compared to the Paúl do Mar population. Similar results were also obtained with other populations of *Ae. aegypti* from the Americas, with transmission ranging between 20–80% [8, 25], and Africa [26]. In the case of a possible chikungunya outbreak on the island, the onset of cases would be alarmingly fast, particularly in Funchal city, where most of the inhabitants live and work.

In addition to CHIKV risk assessment, vector competence for DENV-2 transmission was also evaluated. The reason DENV-2 was chosen for the vector competence study was related to the increasing concern that a new serotype will arrive in Madeira. Dengue secondary infection might lead to severe clinical symptoms and potential fatalities [9]. Our results underline a significantly higher dissemination efficiency of DENV-2 in *Ae. aegypti* from Funchal when comparing to Paúl do Mar. However, we observed no significant differences in transmission rate between the two populations or in the number of virus particles in mosquito saliva. This suggests that higher dissemination of DENV-2 in *Ae. aegypti* may not be correlated with the higher transmission in saliva. It would be interesting to verify if this result can be found with another serotype [27]. Funchal city differs from Paúl do Mar in possessing higher human and *Ae. aegypti* densities favourable to arbovirus transmission as illustrated by the 2012 dengue outbreak caused by DENV-1. Funchal was the central hotspot for DENV-1 transmission, and no DENV-1 cases were observed in Paúl do Mar [23]. Similar viral midgut infection and dissemination rates were observed in other studies with *Ae. aegypti* from the Americas, Australia and, surprisingly, from Africa, even using different methodologies than the one used in this study [7, 28–32].

Madeira Island could be a stepping-stone for the introduction of ZIKV into Europe. The main factors are present: the vector *Ae. aegypti*, imported cases from Brazil and Venezuela [19], and a naïve human population. Vector competence studies for ZIKV were also performed using the same *Ae. aegypti* population described in this study [19]. It has been demonstrated that the Funchal strain was the only population showing viral particles in saliva samples [19]. One should also note that the level of vector competence mostly depends on mosquito population genetics and the viral genotype used in the oral infections [25]. To provide a complete risk assessment of arboviral emergence, more studies should be implemented using additional viral strains or genotypes circulating in areas neighbouring Madeira Island.

CHIKV and DENV are two arboviruses with the highest potential to be introduced to Madeira Island. Based on genetic markers (mtDNA and *kdr* mutations), it has been shown that *Ae. aegypti* from Madeira originated from Brazil or Venezuela [33]. Owing to the extensive exchanges of goods and people with the two South American countries, the risk of CHIKV autochthonous cases on Madeira Island remains high. As previously stated, the Asian genotype of CHIKV was used for the oral infections in *Ae. aegypti* from Madeira. CHIKV has had a severe impact in the Americas since 2014, particularly in Venezuela with the highest number of cases recorded in the Andean region [34]. As with CHIKV, all DENV serotypes can be introduced to Madeira by a viremic traveller returning to Funchal from Caracas [35]. Caracas is connected to the island by weekly direct flights [24].

This study also highlights the need for further studies to define the genetic background of the *Ae. aegypti* populations of Madeira. Differences observed in DENV dissemination between *Ae. aegypti* from Funchal and Paúl do Mar could suggest population-based differences. Attention should be given to differential gene expression related to insecticide resistance [36] or immunity genes which may explain the differences observed. Moreover, the natural habitat of both populations presents distinct environmental and typological conditions: Funchal is considered an urban area, with vector control activities whereas Paúl do Mar is mainly a rural area, geographically isolated from the rest of the island, and with a higher mean temperature along the year. Therefore, the role of environmental and genetic factors should be considered. Additional population genetic studies are being performed with polymorphic DNA markers to refine our knowledge about the origin, genetic differentiation, and stability of the species in the island.

The temperate climate on Madeira Island can also play a key role in modulating *Ae. aegypti* vector competence for arbovirus transmission. It has been shown that temperature affects the vector competence in a tripartite interaction between mosquito genotype, viral genotype, and environment [25, 37]. Considering the Madeira climate, it would be of great importance to assess the vector competence under lower incubation temperature regimes, such as 20 °C, in contrast to the usual incubation temperature of 28 °C.

## Conclusions

Based on our results, we strongly recommend that a robust and strengthened vector surveillance program be maintained on Madeira Island. There is an urgent need for new control strategies since the local *Ae. aegypti* populations are considered resistant to several insecticide classes [36] and this could lead to the complete failure of the vector control programmes. Our results with CHIKV and DENV suggest that it is crucial for Madeira Island to be prepared for more mosquito-borne disease epidemics. If mosquito densities reach levels like those observed during the dengue outbreak in 2012, immediate control measures, such as intensive community-based campaigns or using alternative non-chemical strategies, should be triggered to prevent arbovirus transmission. Our results are also of great importance for European countries where another species, *Ae. albopictus*, has been implicated in the last chikungunya and dengue outbreaks [13–15]. Coordination of vector control strategies between all European countries should be implemented as globalisation will contribute to the growing expansion of vector-borne pathogens, mosquito vectors and viremic people.

## Abbreviations

CHIKV: Chikungunya virus
DENV: Dengue virus
ZIKV: Zika virus
ATP: Adenosine triphosphate
FBS: Fetal bovine serum
dpi: Days post-infection
ffu: Focus forming units
IR: Infection rate
DR: Dissemination rate
TR: Transmission rate
